# Analysis of SARS-CoV-2 RNA-dependent RNA polymerase as a potential therapeutic drug target using a computational approach

**DOI:** 10.1186/s12967-020-02439-0

**Published:** 2020-07-07

**Authors:** Syed Ovais Aftab, Muhammad Zubair Ghouri, Muhammad Umer Masood, Zeshan Haider, Zulqurnain Khan, Aftab Ahmad, Nayla Munawar

**Affiliations:** 1 Center of Agricultural Biochemistry and Biotechnology (CABB), University of Agriculture, Faisalabad, Pakistan; 2 Center for Advanced Studies in Agriculture and Food Security (CAS-AFS), University of Agriculture, Faisalabad, Pakistan; 3 Institute of Plant Breeding and Biotechnology, MNS University of Agriculture, Multan, Pakistan; 4 Department of Biochemistry, University of Agriculture, Faisalabad, Pakistan; 5 Department of Chemistry, United Arab Emirates University, Al-Ain, UAE

**Keywords:** RdRp, SARS-CoV-2, Phylogenetic tree, Homology modeling, Molecular Docking, Active site

## Abstract

**Background:**

The Severe acute respiratory syndrome-related coronavirus 2 (SARS-CoV-2) outbreak originating in Wuhan, China, has raised global health concerns and the pandemic has now been reported on all inhabited continents. Hitherto, no antiviral drug is available to combat this viral outbreak.

**Methods:**

Keeping in mind the urgency of the situation, the current study was designed to devise new strategies for drug discovery and/or repositioning against SARS-CoV-2. In the current study, RNA-dependent RNA polymerase (RdRp), which regulates viral replication, is proposed as a potential therapeutic target to inhibit viral infection.

**Results:**

Evolutionary studies of whole-genome sequences of SARS-CoV-2 represent high similarity (> 90%) with other SARS viruses. Targeting the RdRp active sites, ASP760 and ASP761, by antiviral drugs could be a potential therapeutic option for inhibition of coronavirus RdRp, and thus viral replication. Target-based virtual screening and molecular docking results show that the antiviral Galidesivir and its structurally similar compounds have shown promise against SARS-CoV-2.

**Conclusions:**

The anti-polymerase drugs predicted here—CID123624208 and CID11687749—may be considered for in vitro and in vivo clinical trials.

## Background

Severe acute respiratory syndrome coronavirus (SARS-CoV) is a positive-sense single-stranded RNA virus from the genus Betacoronavirus, commonly known to infect bats, humans, and other mammals [1–4]. On January 30th, 2020, the Director-General of the World Health Organization (WHO) declared that the outbreak of novel coronavirus (2019-nCoV) constitutes a Public Health Emergency of International Concern (PHEIC). By April 10th, 2020, the current pandemic caused by the 2019-nCoV has reached nearly all the world’s countries and has consisted of more than 1.5 million confirmed cases with more than 92,000 deaths [5]. To date, two SARS strains have been reported to cause epidemics: (1) SARS-CoV, identified in 2002–2004, and (2) SARS-CoV-2, also known as the novel coronavirus that emerged as a potential threat in late 2019 [6]. Both these strains evolved from a common Betacoronavirus ancestor; however, it is believed that SARS-CoV-2 first infected humans from a bat host during interspecies viral transmission. In support, it has been reported in China and other countries that bats are the primary reservoirs of SARS-CoV-2 [6–8]. Coronaviruses are a large family of viruses reported to cause illnesses ranging from the common cold to severe diseases such as Middle East respiratory syndrome (MERS) and severe acute respiratory syndrome (SARS). SARS-CoV-2 is one of the seven coronaviruses known to cause infection in *Homo sapiens*, which also includes: 229E (HCoV-229E), NL63 (HCoV-NL63), OC43 (HCoV-OC43), HKU1 (HCoV-HKU1), MERS-CoV, and the original SARS-CoV [9–12].

The novel coronavirus is the most relevant virus of the family Coronaviridae in terms of research currently, as there remains no approved antiviral drug or vaccine against it [13]. Recently, on the basis of genomic resemblance with previously reported SARS-CoV, the International Committee on Taxonomy of Viruses (ICTV) coronavirus study group named this virus “SARS-CoV-2” [13]. It has been confirmed that SARS-CoV-2 can spread with human-to-human transmission via respiratory droplets (e.g. through coughing or sneezing) or even by contact with contaminated surfaces [14, 15]. A coronavirus epidemic was previously predicted by the WHO soon after the Ebola virus outbreak in 2016 [16, 17]; and this prediction came to fruition in the Wuhan city seafood market with the coronavirus epidemic of 2019–2020 [18–20]. Therefore, scientists are attempting to use preexisting antiviral drugs to control the virus upsurge, however, these drugs have thus far had inappreciable effects on SARS-CoV-2 [21, 22]. Efficacy of such antiviral drugs may be compromised due to changes induced by single nucleotide polymorphisms (SNPs), thereby resulting in amino acid shifts which ultimately modify functional viral protein(s). This could be the case for SARS-CoV-2, in that viral proteins are actively acquiring mutations due to SNPs and thus escape from being targeted by antiviral drugs [13, 23, 24].

Genome organization of all coronaviruses are similar and contain 5′ and 3′ untranslated regions (UTR’s) for characteristic genes coding for ORF1ab, spike, envelope, membrane, and nucleocapsid proteins [24]. ORF1ab is of particular importance, as it occupies two-thirds of the CoV genome and encodes a replicase polyprotein from ORF1a and ORF1b. In addition, a slippery sequence (UUUAAAC) is present at the junction between ORF1a and ORF1b in all coronaviruses, with translation commencing at the end of slippery sequence via a − 1 RNA-mediated ribosome frame shift [25–27]. Papain-like protease (PLpro) and 3C-like protease (3CLpro) are proteins encoded by ORF1ab and cleave the replicase polyprotein into 15–16 non-structural proteins (nsps) at consensus cleavage sites. Some of these nsps encode proteins with essential functions, such as PLpro (nsp3), 3CLpro (nsp5) and RdRP (nsp12) which plays an important role in viral replication, whereas helicase (nsp13) has been recognized to unwind duplex oligonucleotides in an NTP-dependent manner [26–28].

Most RNA viruses—except for retroviruses—require an RdRp for replication and transcription of the viral genome, making it essential for their survival [29, 30]. The RdRp protein ranges from 240 to 450 kD and consists of a catalytic core with a clear resemblance to the human right hand with differentiated palm, fingers and thumb domains [30, 31]. RdRp is considered to be a conserved protein within RNA viruses, and thus could be used as an attractive candidate for understanding their biology in terms of nucleic acid synthesis and development of antiviral drugs [32–39]. RdRp plays a crucial role in the viral life cycle, and as the active site of the RdRp is the most conserved and accessible region, targeting this region for inhibition of viral replication may be an effective therapeutic approach. Scientists worldwide have proposed the use of preexisting drugs against the novel coronavirus; however, the efficacy of these drugs is somewhat limited. Others reported repurposing the use of existing antiviral agents in order to reduce time and cost compared to de novo drug discovery [40]. Ribavirin, lopinavir–ritonavir, corticosteroids, and interferon are just a few of the antiviral drugs that have been tested for use against SARS-CoV-2 [41, 42].

Soon after the COVID-19 outbreak, China initiated several in vitro studies and clinical trials to discover effective antiviral drugs. Favipiravir is the first antiviral drug that has been approved by National Medical Products Administration of China to use against SARS-CoV-2 [43]. A number of drugs—such as Sofosbuvir, Ribavirin, Galidesivir, Remdesivir, etc.—are in clinical trials for different viruses (e.g. Hepatitis C, Zika virus, Dengue and SARS-CoV-2) on the basis of anti-RdRp activity [44–51]. The core objective of the current study was to evaluate the potential of Galidesivir, similar to structurally similar antiviral compounds, to inhibit the viral replication protein RdRp. An evolutionary-based study was performed to understand the relationships of SARS-CoV-2 genome with other SARS isolates. Homology modeling for predicting SARS-CoV-2 RdRp structure was performed with a reference template of SARS-CoV RdRp. Among the eight antiviral agents examined (Ribavirin, penciclovir, nitazoxanide, nafamostat, chloroquine, Galidesivir, favipiravir, and interferon), the best suited inhibitors were screened through in silico analysis that can further be used for preclinical trials to halt viral replication after prior testing. Our results are promising and may be considered for both in vitro and in vivo clinical trials for inhibition of COVID-19.

## Materials and methods

### Sequence retrieval, alignment and evolutionary relationship of viral species

The SARS-CoV-2 whole-genome sequence was retrieved from NCBI (National Center for Biotechnology Information) under Accession # NC_045512.2. In addition, a total of 94 whole-genome viral sequences belonging only to class Nidovirales, order Coronaviradae, family Coronavirinae, and three genera (Alphacoronavirus, Betacoronavirus, and Gammacoronavirus) were downloaded from the GenBank database for sequence similarity comparison with our experimental sequence of SARS-CoV-2. The sequences were submitted to MEGA v7 in FASTA format, aligned using CLUSTALW alignment tool and maximum likelihood estimation (MLE) tree was constructed against all viral genome sequences for tracing the evolutionary record with SARS-CoV-2 using a bootstrap value of 1000.

### Protein sequence retrieval and estimation of physicochemical properties

Sequence of RdRp protein for SARS-CoV-2 (YP_009725307.1) was retrieved from the NCBI protein database and contains 932 amino acids (AA). In addition, physicochemical properties—such as molecular weight, amino acid composition, atomic composition, extinction coefficient, estimated half-life, instability index, aliphatic index and grand average of hydropathicity (GRAVY)—were analyzed through ProtParam tool of ExPASy for evaluation of the primary structure of RdRp protein.

### Homology modeling and protein phylogenetic analysis

RdRp protein AA from SARS-CoV and SARS-CoV-2 were aligned with the help of Clustal X 2.1 software using default parameters, and the sequences were further refined by GeneDoc to find the similarity between them. This similarity helps to select a template for modeling and further analysis. RdRp proteins of SARS-CoV [32] and novel SARS-CoV-2 were compared for their amino acid residues. Based on similarity in amino acid residues of both RdRps, a reference template of SARS-CoV RdRp (PDB ID: 6NUR; kindly provided by the Ward lab) was used for further analysis [52]. Homology modeling was performed with the MODELLER v9.1 program using 6NUR as reference and ~ 100 structures were predicted [53]. The best possible structure was selected based on highest discrete optimized protein energy (DOPE) score. In addition, this best suited structure was placed in a Ramachandran plot for evaluation of chemical properties, bonds, and angles of RdRp. Moreover, the RdRp protein sequence of SARS-CoV-2 was subjected to pBLAST to infer its similarity with other RdRp protein sequences; 94 total sequences were selected that exhibited > 90% similarity with our input RdRp sequence. Likewise, these 94 validated sequences were uploaded in MEGA v7 and further aligned through the CLUSTALW alignment tool. Aligned sequences were used to construct a MLE tree for understanding the phylogenies of all the validated protein sequences along with SARS-CoV-2 RdRp.

### Ligand selection and virtual screening

For docking, the following ten antiviral drugs were screened: Ribavirin, Remdesivir, Sofosbuvir, Penciclovir, Nitazoxanide, Nafamostat, Chloroquine, Galidesivir, Favipiravir, and Interferon. Docking was performed using molecular operating environment (MOE) software. Based on the phylogenetic similarity of the RdRp proteins of SARS-CoV and SARS-CoV-2, the catalytic domain was selected for inhibitor docking of inhibitors against the SARS-CoV-2 RdRP. Antiviral drugs were docked against the following catalytic sites: GLY616, TRP617, ASP618, TYR619, LEU758, SER759, ASP760, ASP761, ALA762, LYS798, TYS799, TRP800, GLU811, PHE812, CIS813, and SER814. The best antiviral drug was selected with distinct S-score, root-mean-square deviation (RMSD), and energy binding score for the catalytic domain of RdRp SARS-CoV-2. The pre-eminent ligand was screened against the PubChem database for detection of drug-like compounds, and the Pfizer rule was applied to evaluate drug-like properties. Different parameters for drug evaluation were considered, such as: molecular weight (MW) < 500, LogP < 5, H-bond doner(s) < 10, and H-bonding accepter(s) < 5. Finally, compounds fulfilling the above-mentioned requirements were used for molecular docking into a new database.

### Molecular docking

All the retrieved compounds were docked using selected catalytic site of the three-dimensional structure of RdRp protein. Removal of water, 3D protonation and energy minimization were carried out with the MOE software using the following parameters: force field, MMFF94X, gradient 0.05, and current geometry [54, 55]. The binding pocket of the RdRp—GLY616, TRP617, ASP618, TYR619, LEU758, SER759, ASP760, ASP761, ALA762, LYS798, TYS799, TRP800, GLU811, PHE812, CIS813, and/or SER814—was found using the MOE SiteFinder algorithm, and most optimal docked compounds were selected based on higher S-score and lower RMSD with a reference inhibitor (PubChem CID: 10445549). Docking simulations were performed for Galidesivir, a potential non-covalent inhibitor of SARS-CoV-2 RdRp, which binds strongly to the active site of RdRp. It also targets the ASP761, ALA762, LYS798, and SER814 residues with stout hydrogen bonding and a maximum docking score. For the discovery of novel drug-like compounds, we screened compounds structurally similar to Galidesivir that may target SARS-CoV-2 RdRp as an antiviral agent. The best hits—CID123624208 and CID11687749—were selected from the docking analysis.

### AdmetSAR profiling and toxicity validation

Virtually-selected drug-like compounds were used in admetSAR online tool (Immd.ecust.edu.cn/admetsar2) to evaluate their level of toxicity. This program predicts toxic effects—such as carcinogenicity, mutagenicity, irritant effect, reproductivity and drug-like physical and chemical properties of compounds—which can help for selection of compounds that can be used as safe antiviral agents on humans.

## Results

### Evolutionary relationship of SARS-CoV-2 with other viral isolates

**Fig. 1:**
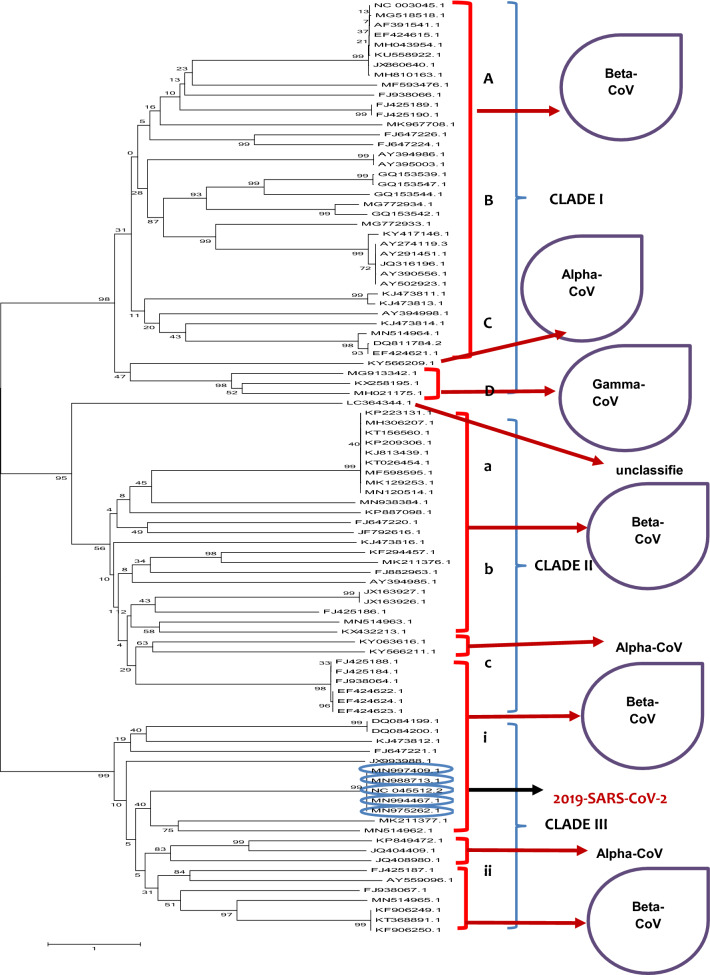
Dendrogram representing the phylogeny of viruses with SARS-CoV-2. Phylogenetic tree is divided into three clades, and all clades are further divided into sub-groups. 2019-SARS-CoV-2 from clade III is closely related to its relatives from Hong Kong and USA (circled in blue). For accessions, origin and other relevant data, see Additional file 1

A phylogenetic tree infers relative evolutionary distance with respect to other species. Tracing evolutionary history among all possible viral relatives using a genome sequence of interest can elucidate the adaptive behavior of species. In this study, we identified interspecies divergence among all the 94 validated viral genomic sequences belonging to the family Coronavirinae retrieved from the NCBI database. Most of the species clustered together, reflecting prior taxonomic identification, with a few exceptions. Overall, the phylogenetic tree was divided into three different clades (Fig. 1). The accessions, origin and taxonomic classification of all sequences are given in Additional file 1. Most of the accessions in clade I belong to the Betacoronaviruses, indicating they all are closely related to each other, while only one and two accessions belong to alpha-CoV and gamma-CoV, respectively, thereby representing some distance with respect to the Betacoronavirus members. Clade II also included most of the beta-CoV members, with only two viral species clustered in a separate clade belong to alpha-CoV. Most importantly, clade III—which included the experimental SARS-CoV-2 isolate originating from the Wuhan seafood market—was found in close relation with all the other isolates of SARS-CoV-2 reported from Hong Kong and the USA (MN975262.1, MN997409.1, MN994467.1, MN988713.1; Fig. 1).

### Estimation of physiochemical properties

The feasibility of protein structure depends upon three-dimensional conformation based on amino acid sequence. Protein sequences were assembled based on their physicochemical properties. ProtParam (ExPASy) results for physicochemical properties revealed that the RdRp polypeptide contains 932 AA with a molecular weight of 106,660.24 Da, GRAVY score of − 0.224, an instability index of 28.32, and is capable of making hydrogen bonds, categorizing it as a stable protein (Table 1).

**Table 1:** Physicochemical properties of RdRP protein (YP_009725307.1)

Physiochemical properties	RdRP COVID-19	Amino acid composition	No.	Percent composition (%)
Molecular weight	106,660.24	Ala (A)	64	6.90
No. of amino acids	932	Arg (R)	43	4.60
Theoretical pI	6.14	Asn (N)	56	6.00
Instability index	28.32	Asp (D)	75	8.00
No. of negatively charged (Asp + Glu)	106	Cys (C)	29	3.10
No. of positively charged (Arg + Lys	94	Gln (Q)	28	3.00
aliphatic index	78.43	Glu (E)	31	3.30
Grand average of hydropathicity	−0.224	Gly (G)	45	4.80
Estimated half-life (mammalian reticulocytes, in vitro)	1.9 h	His (H)	27	2.90
Atomic composition		Ile (I)	33	3.50
C	4792	Leu (L)	83	8.90
H	7265	Lys (K)	51	5.50
N	1259	Met (M)	25	2.70
O	1401	Phe (F)	57	6.10
S	54	Pro (P)	30	3.20
Formula	C_4792_H_7265_N_1259_O_1401_S_54_	Ser (S)	53	5.70
Total number of atoms	14,771	Thr (T)	61	6.50
		Trp (W)	9	1.00
		Tyr (Y)	58	6.20
		Val (V)	74	7.90
		Pyl (O)	0	0.00
		Sec (U)	0	0.00

### Modeling and structure prediction

**Fig. 2:**
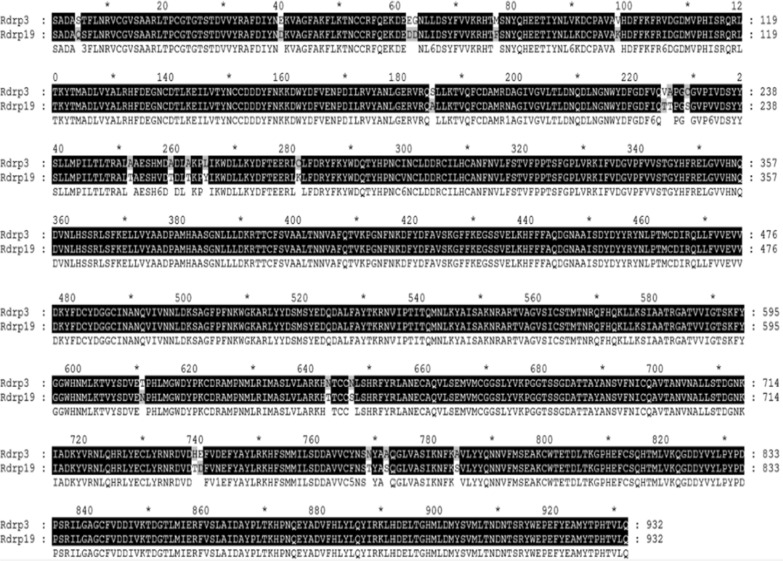
RdRp Sequence alignment of SARS-CoV and SARS-CoV-2. RdRp sequences of SARS-CoV and SARS-CoV-2 were aligned for analysis of divergence, the dark region represents the conserved regions and lighter regions highlight changes in amino acids in specific sites. Only 2.46% divergence was observed between the two RdRp protein sequences

**Fig. 3:**
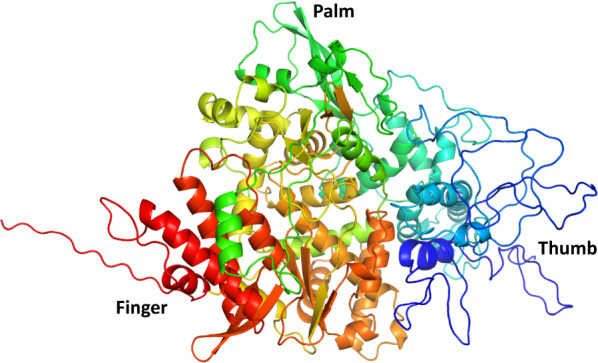
3D structure of SARS-CoV-2 RdRp using reference template (6NUR) predicted through PyMol9.19. The subdomains of RdRp are represented as follows: palm domain in green, thumb domain in blue, and finger domain in red

**Fig. 4:**
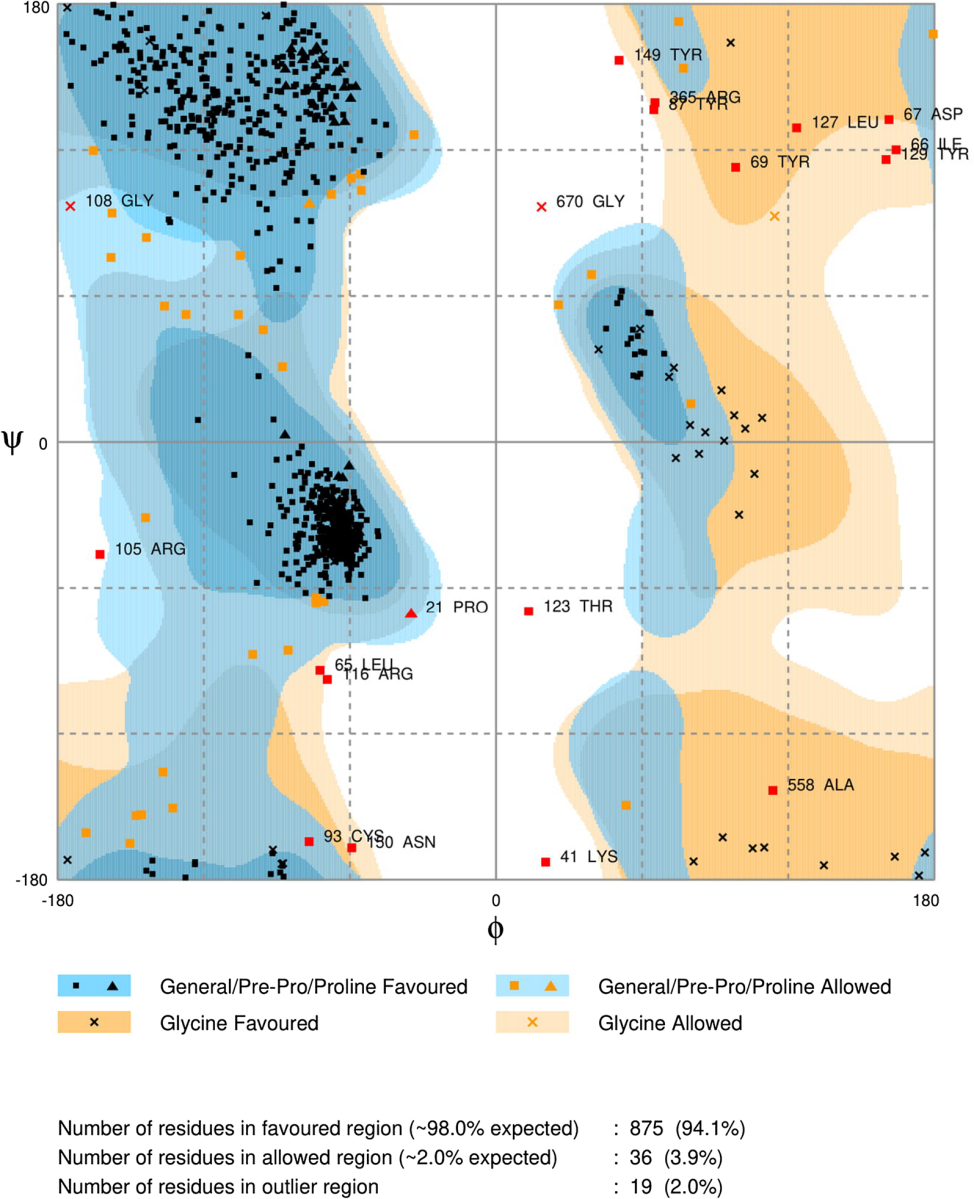
Ramachandran plot for the model of RdRP SARS-CoV-2 protein representing 98% residues in most favored region, ~ 2% in allowed regions while 2% residues in outlier regions

Results of comparison of amino acid residues demonstrated that the RdRps of SARS-CoV and SARS-CoV-2 are mostly conserved. Overall, divergence was observed in only 23 out of 932 AA (Fig. 2). According to our results, 2.46% divergence was observed, thus we selected the SARS-CoV RdRp (6NUR) protein structure as a reference template for the purpose of homology modeling. The protein sequence of SARS-CoV-2 RdRp (YP_009725307.1) was retrieved from NCBI and submitted to a protein databank for PSI-BLAST (https://www.rcsb.org/), which depicts 96% similarity with the 6NUR protein [52]. Using the 6NUR protein structure as a template, 100 models were generated with MODELLER and the SARS-CoV 3D structure was selected with a DOPE score of -108,832.65625 and GA score 1 (Fig. 3). RAMPAGE Ramachandran plots indicate that most of the amino acids are within favorable regions (875 AA), making up 94.1% of the expected residues. There was no need for refinement analysis, as the Ramachandran plot shows 94.1% in most favored regions. Moreover, outlier AA represented on the plot shows the desired AA which are to be targeted by our ligand reside in favored regions (Fig. 4).

**Fig. 5:**
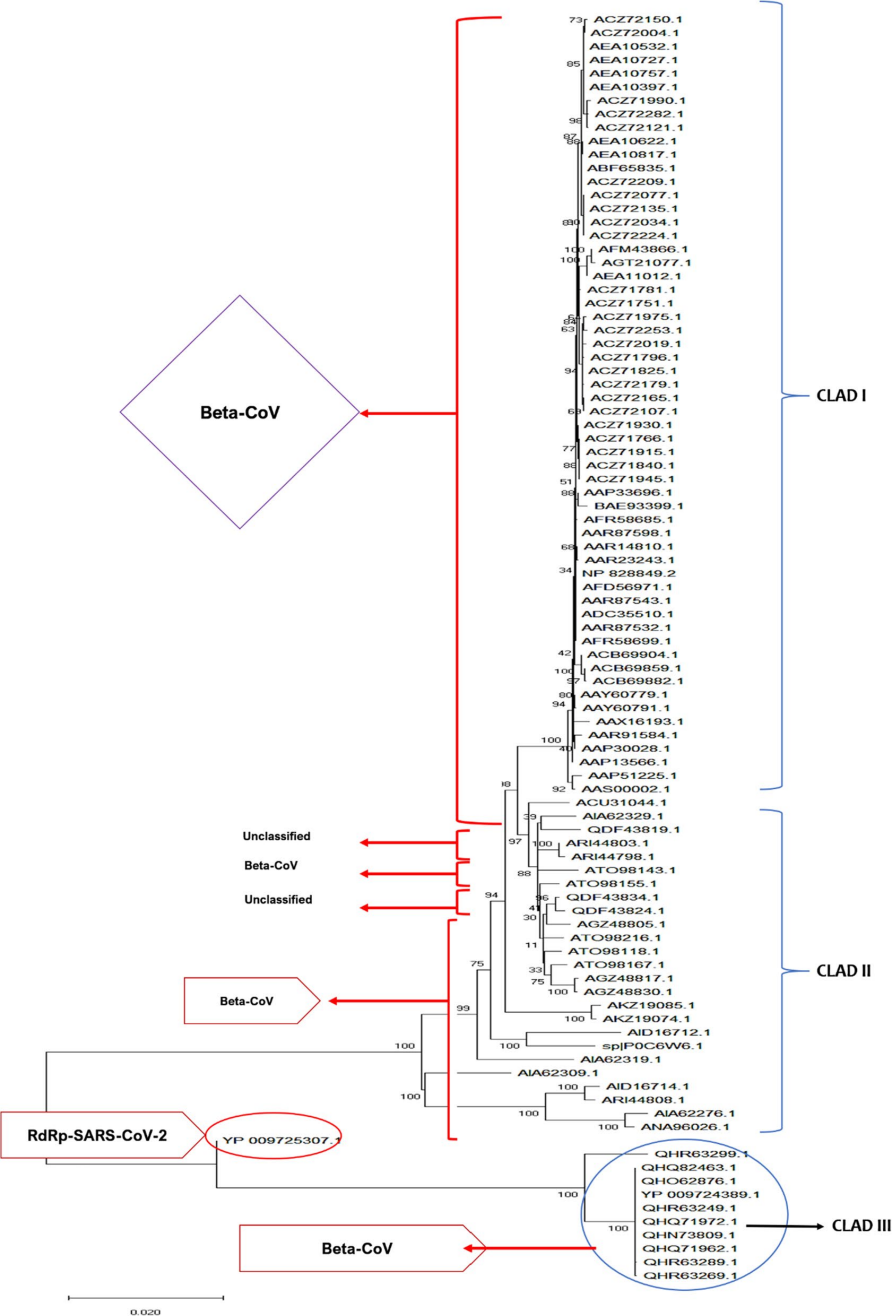
Dendrogram representing evolutionary relationship of RdRp proteins of SARS-CoV-2 with other RdRp proteins. SARS-CoV-2 accession (red circle) is closely associated with other SARS RdRp proteins (blue circle). For accessions, origin and other relevant data, see Additional file 1: Data 2

The SARS-CoV-2 RdRp protein sequence was queried using pBLAST and 94 validated sequences were downloaded from the NCBI database for evaluating its phylogenetic relationship with other viral RdRp proteins. An MLE tree constructed for all validated proteins revealed that most of the proteins shared close association with each other. Overall, the RdRp protein tree was divided into three clades: I, II, and III. Most of the viral RdRps in clade I belong to beta-CoV; the same is true for clades II and III with most accessions belonging to beta-CoV, while a few remain unclassified and require more research for proper designation. Accession QHR63299.1 was also unclassified but shows close association with other SARS RdRp relatives (Fig. 5).

### Ligand selection and virtual screening

All the selected ligands from a previous literature survey were docked with the RdRp protein of SARS-CoV-2. The docking score, RMSD, and binding energy of ligands with receptor RdRp are shown in Table 2. The results demonstrated that all the ligands exhibit interaction with the pocket of RdRp, while, Remdesivir has the highest S-score and lowest binding energy with the active pocket of RdRp of SARS-CoV-2. However, in virtual screening, Remdesivir and Sofosbuvir do not provided us with novel compounds against SARS-CoV-2 RdRp. Therefore, we have selected Galidesivir with S-score (− 12.5338) and binding energy (− 13.922) that provided us with some novel compounds in virtual screening (Table 3). Compounds with > 95% structural similarity to Galidesivir were selected for ligand-based virtual screening from the PubChem database. A total of 1061 compounds were retrieved and Pfizer’s rule of five was applied to all the compounds, out of which 677 were aligned after minimizing energy in a new database for docking with the protein of interest RdRp.

**Table 2:** Docking scores of selected ligands against RdRp SARS-CoV-2

Sr. no	Inhibitor	S-score	RMSD
1.	Remdesivir	− 14.06038	2.357701
2.	Galidesivir	− 12.5338	1.0217
3.	Ribavirin	− 11.8884	0.9554
4.	Sofosbuvir	− 11.132	2.08
5.	Penciclovir	− 10.9772	0.9461
6.	Chloroquine	− 10.3804	1.6397
7.	Nitazoxanide	− 9.7636	2.4603
8.	Nafamostat	− 9.76403	2.4605
9.	Interferon	− 9.62582	1.7336
10.	Favipiravir	− 9.36643	1.2231

**Table 3:** Docking score, binding energy, and Lipinski’s rule scan results for selected compounds

PubChem ID	S-score	RMSD	Binding energy kcal/mol	Lipinski’s drug-likeness score
CID123624208	− 12.2589	0.4971	− 20.812	MW = 309.282, LogP = − 1.247, H-bond donors = 6, H-bond acceptors = 4, and tPSA = 168.820
CID11687749	− 12.7751	0.9957	− 19.601	MW = 279.30, LogP = − 1.247, H-bond donor = 6, H-bond acceptor = 4, tpSA = 140.31

### Docking simulations

**Fig. 6:**
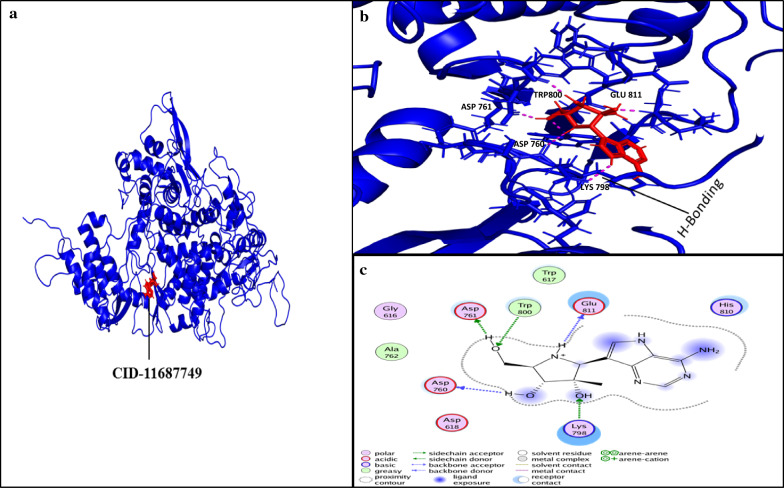
Ligand interaction with conserved amino acid residues of RdRp. **a** RdRp-ligand (CID-11687749; red) inhibitor complex. **b** Ligand conformation in active site of RdRp; the pink dotted line represents H-bonding between the amino acid residues of RdRp and ligand. **c** 2D representation of ligand interaction with receptor (RdRp). H-bonding of residues ASP760, ASP761, GLU811, TRP800, and LYS798 of RdRp with inhibitor CID-11687749. **a** and **b** were analyzed with PyMol 9.1 while **c** was analyzed using MOE software

**Fig. 7:**
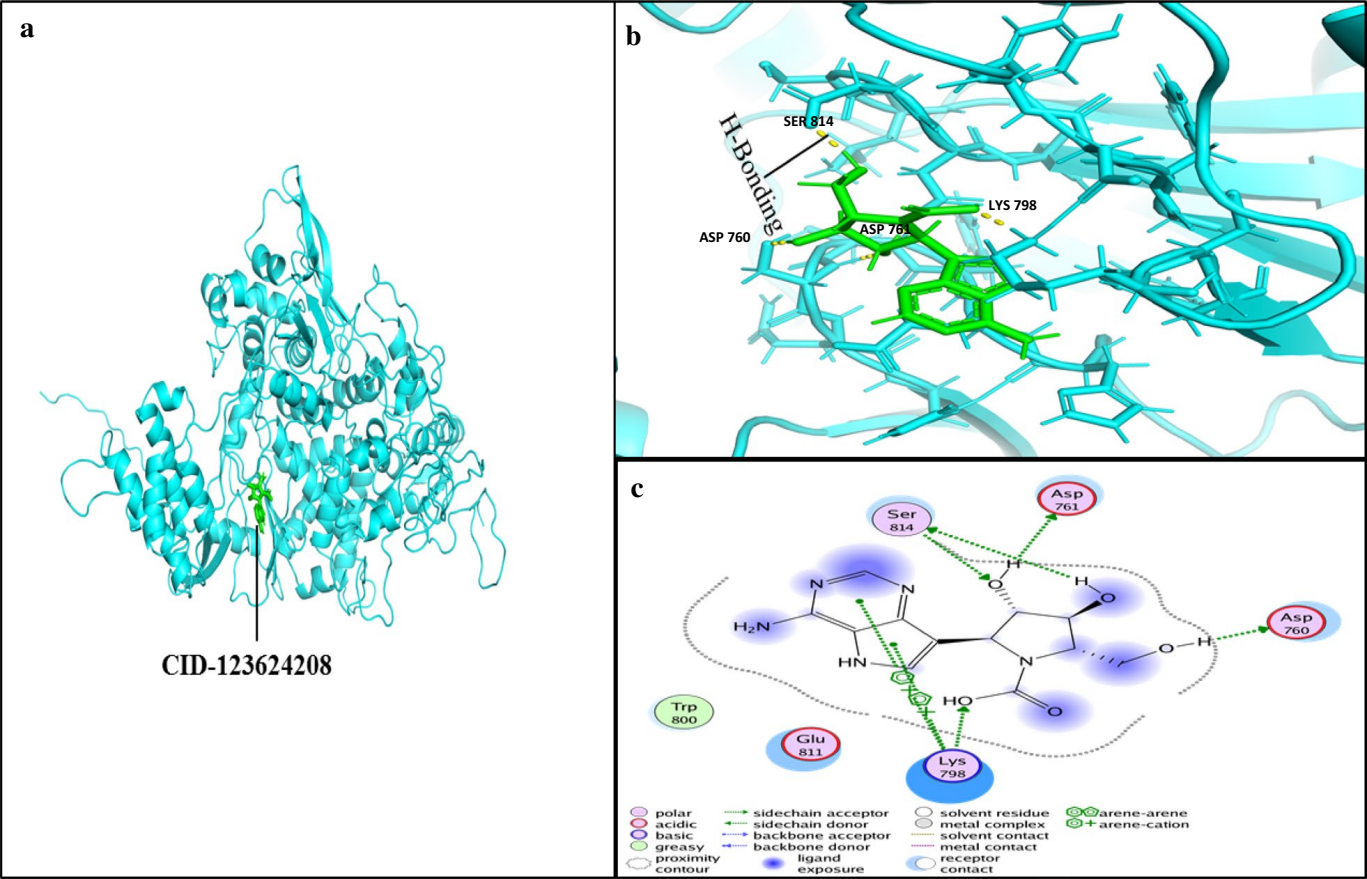
Ligand interaction with conserved amino acid residues of RdRp. **a** RdRp-ligand (CID-123624208; green) inhibitor complex. **b** Ligand conformation in active site of RdRp; yellow dotted line represents H-bonding between the amino acid residues of RdRp and ligand CID-123624208; (green). **c** 2D representation ligand interaction with receptor (RdRp). H-bonding residues ASP760, ASP761, SER814, and LYS798 of RdRP with inhibitor (CID-123624208) are shown with the green line. **a**, **b** were analyzed with PyMol 9.1 while **c** was analyzed using MOE software

**Fig. 8:**
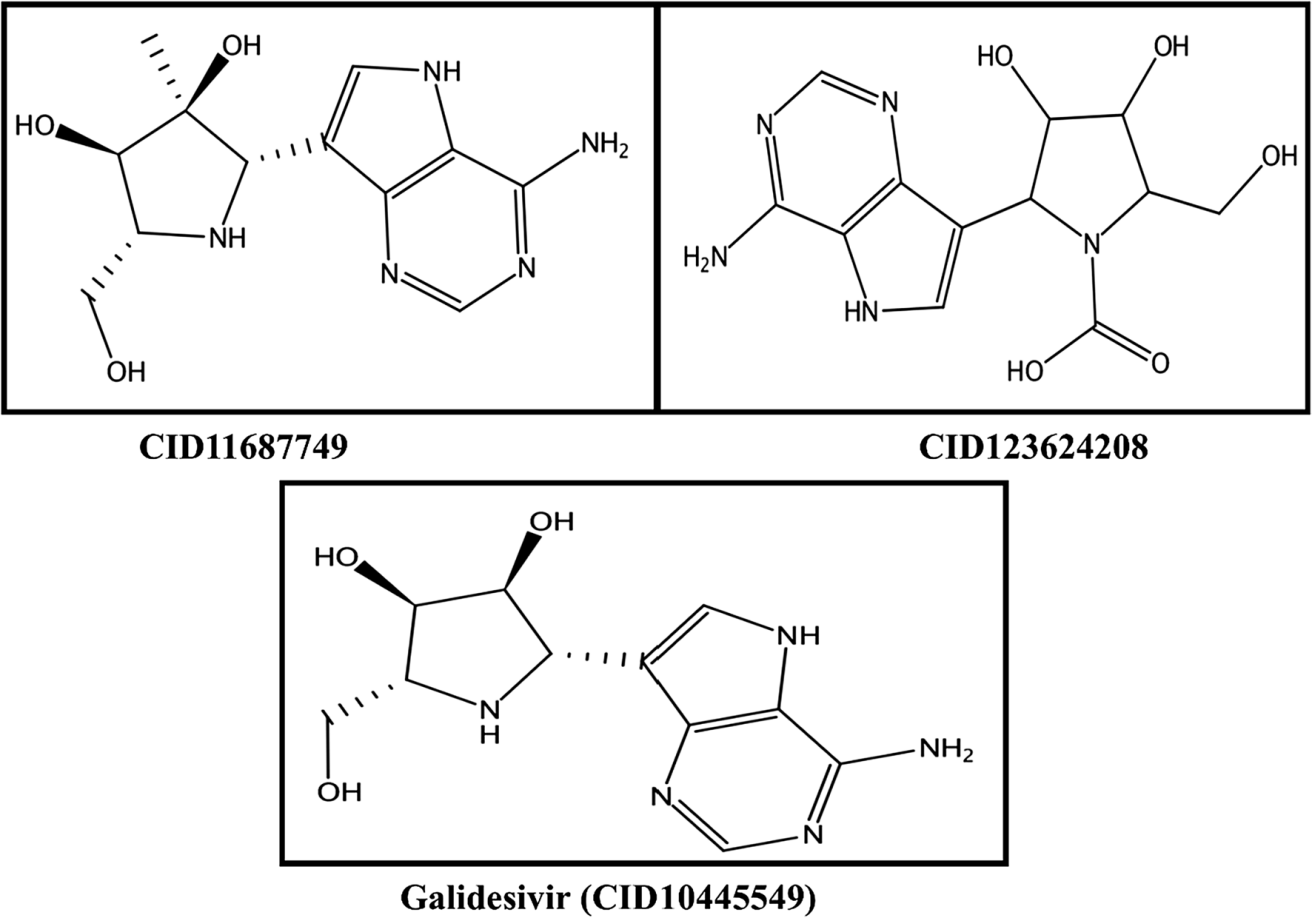
2D structure of selected inhibitor with respective PubChem IDs

Docking simulations demonstrated that the ligand Galidesivir strongly binds within active sites containing the following residues: ASP760, ASP761, GLY616, TRP617, ASP618, TYR619, PRO620, LYS621, CYS622, LEU758, SER759, ALA762, ALA797, LYS798, CYS799, TRP800, HIS810, GLU811, PHE812, CYS813, SER814, and GLN815. Of these, the sites ASP761, ALA762, LYS798, and SER814 strongly bind Galidesivir, while the other AA found in close vicinity were involved in making the interface of ligand and receptor complex (Fig. 6). CID123624208 and CID11687749 interactions with RdRP are presented in Figs. 6 and 7. We suggest two non-toxic drug-like compounds predicted to bind ASP761, ASP760, SER814 and LYS798 sites (Figs. 6, 7) through stout hydrogen bonding. ADMET profiling for drug-like compounds demonstrated positive results for blood–brain barrier (BBB), human intestinal absorption (HIA), renal organic cation transporter (ROCT) for CID11687749 and CID123624208 (Table 3), whereas CaCO_2_ permeability results were found to be negative for both the compounds. Ames toxicity testing results revealed that the compounds were non-toxic and non-carcinogenic (Table 3, Fig. 8). CID123624208 and CID11687749 were the best suggested drug-like compounds analyzed through in silico analysis and may act as strong inhibitors against SARS-CoV-2. We suggest that these novel compounds could be used for preclinical trials as antiviral agents against SARS-CoV-2.

## Discussion

Sequencing of novel viruses such as SARS-CoV-2 helps to identify target regions for intervention. Scientists took less than 60 days to trace the origin of SARS-CoV-2, however, this virus may show similarity to other viruses based on nucleotide sequences, such as MERS, which could help to find attractive therapeutic options [53, 54, 56, 57]. It has been reported that nucleotide sequence-based analysis of SARS-CoV-2 and SARS-CoV (Bats origin) represent a close relation with each other but sufficiently divergent due to single nucleotide mutations [13, 21, 23, 56, 57]. In our study, 94 validated sequences were used for construction of a phylogenetic tree to trace out similarity between all the sequences from alpha-, beta-, and gamma coronaviruses. Most of the viral species examined showed resemblance to each other and clustered together. SARS-CoV-2 whole-genome sequences reported from Hong Kong and USA (MN975262.1, MN997409.1, MN994467.1, MN988713.1) revealed close relation with each other as they originated from same clade and are considered as sister species (Fig. 1). Our results are consistent with the results of Wu et al. [58], who demonstrated that SARS-CoV-2 and bat-origin SARS-CoV clustered together, revealing similarity in their genomes (Fig. 1).

Physicochemical properties of the RdRp protein used in our study revealed satisfactory results in terms of molecular weight, GRAVY score, and instability index capable of making strong hydrogen bonds, and are similar to previous data reported by Dwivedi et al. [59]. Homology modeling results within the current study are consistent with those reported by Xu et al. [32] and Sarwar et al. [60]. However, the latter group only predicted 10 models, instead of the ~ 100 structures here. In addition, of these possible structures we selected the most suitable among them to predict the 3D structure of the RdRp protein of SARS-CoV. Moreover, in our study, the RdRp protein sequence was searched for similarity with other viral RdRps. Our results demonstrated that all the SARS-CoV-2 RdRp proteins relatively clustered together, which included isolates from Hong Kong, USA and Wuhan (Fig. 5). It is suggested that the close association between all these RdRps is due to the similarity in their genome organization. Therefore, more research is needed to explore the differentiation between these viral RdRps.

**Fig. 9:**
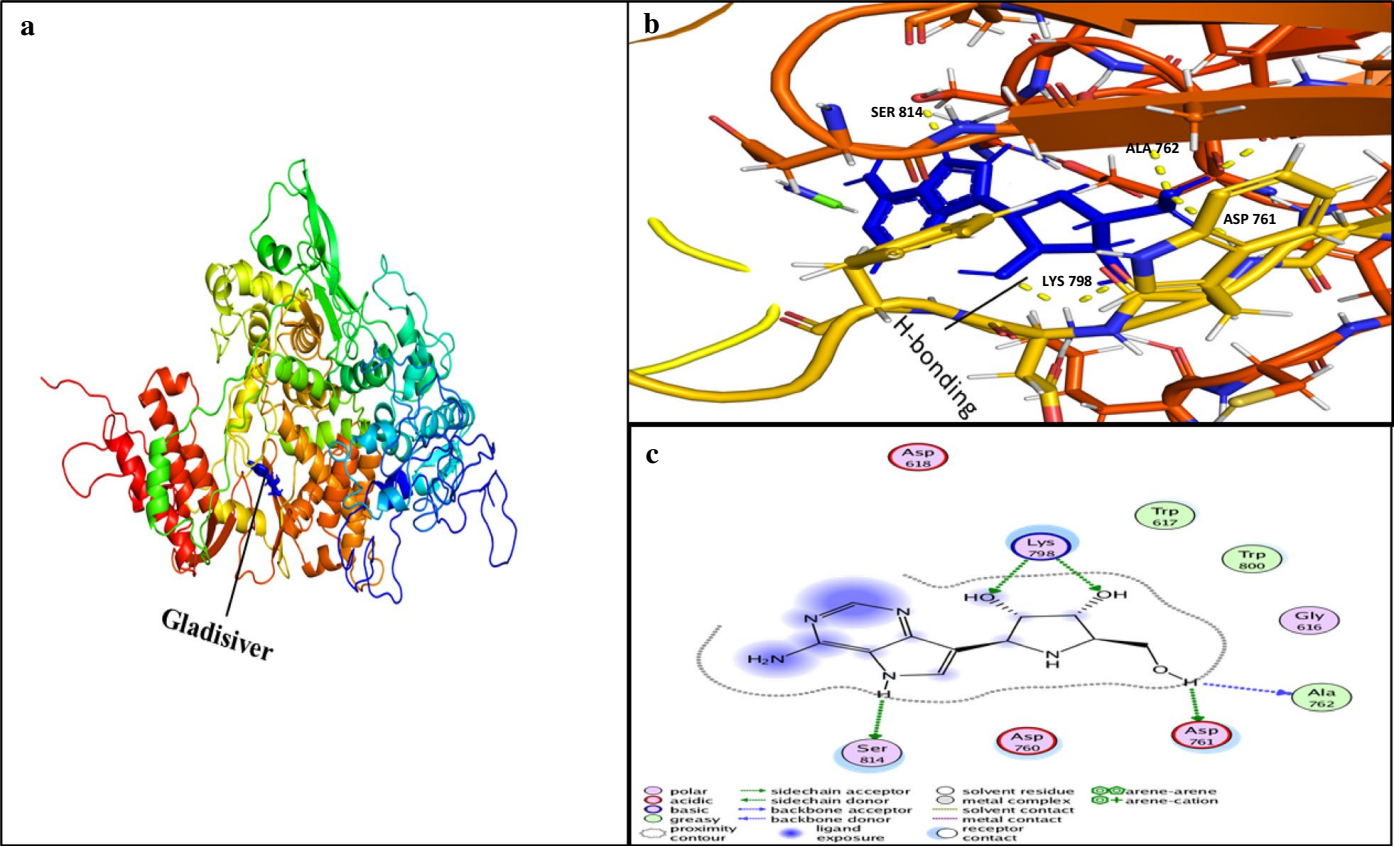
Ligand interaction with conserved amino acid residues of RdRp. **a** RdRp-Galidesivir inhibitor complex, Galidesivir represented in blue color. **b** Shows the ligand interaction in active site of RdRp. Yellow doted lines represent the H-Bonding (hydrogen bonding) between the amino acid residues of RdRp and ligand Galidesivir (blue color). **c** 2D representation of ligand interaction with receptor (RdRp). H-bonding residues ASP-761, ALA-762, LYS-798, and SER-814 of RdRp with inhibitor Galidesivir are shown in green dotted line. **a** and **b** were analyzed by PyMol 9.1 while **c** was analyzed by MOE

Docking simulations of results of our study are in line with Xu et al. [32] regarding their strong interaction with the following active sites: ASP760, ASP761, GLY616, TRP617, ASP618, TYR619, PRO620, LYS621, CYS622, LEU758, SER759, ALA762, ALA797, LYS798, CYS799, TRP800, HIS810, GLU811, PHE812, CYS813, SER814, GLN815 (Figs. 6, 9). This group also reported that ASP760 and ASP761 are responsible for composing the RdRp catalytic domain, while SER814 is involved in positioning of the priming nucleotide, and our results are consistent with the previous studies [60–64]. Similarly, the LYS798 residue was found to be involved in stabilizing the core structure of the RdRp domain, the finding is in line with other studies [63–65]. In a recent study, Gao et al. reported that nucleoside analogs such as Remdesivir and Sofosbuvir strongly binds with ASP760, ASP761 and ASP618 residues to inhibit RdRp of SARS-CoV-2 [66]. Our study is similar to Gao et al. reporting nucleoside analogs can form hydrogen bonds with active site of RdRp.

Our study indicates that the novel predicted drug-like compounds CID123624208 and CID11687749 have a strong affinity with the residues of the RdRp catalytic domain (Figs. 6, 7). Strong S-score, binding energy, and RMSD values suggest that these compounds could be used as potential inhibitors against the RdRp of SARS-CoV-2. Numerous other viral inhibitors have also been reported which are in clinical trials including Remdesivir, Sofosbuvir Galidesivir etc., for which we have already tested with virtual screening [67]. However, some of them are stated in Table 2 with their S-score and RMSD values. Thus, in summary, Galidesivir and the two drugs-like compounds CID123624208 and CID11687749 screened in the present study could more likely have potential as therapeutic drugs targeting SARS-CoV-2.

Use of FDA approved anti-polymerase drugs has been the objective of virologists in treating the patients of new viral infection caused by SARS-CoV-2. The choice of FDA approved drugs was a wise decision in this current emergency arises due to COVID-19 pandemic; since they were previously tested before approval by the FDA. The present study is aimed at finding the best alternative drugs to inhibit the RdRp of SARS-CoV-2. Galidesivir and its suggested compounds pose no toxicity and are safe to consume proved in our in silico analysis. The only limitation of the study is to confirm the results of this study using in vivo and in vitro analysis. In the future, further research is direly needed to validate the results of this in silico evaluation for preclinical trials of these potent drug-like compounds CID123624208 and CID11687749.

## Conclusion

The rapidly growing coronavirus cases urge the development of new therapeutics and vaccines against SARS-CoV-2. In the previous studies so far, RdRp-CoV (nsp12) is the most frequently used and suggested potential target to inhibit viral replication. In this study, we tested some important anti polymerase drugs, whether they are currently in clinical trials or in the market to stop viral infection on an emergency basis. However, Galidesivir and its drug-like compounds CID123624208 and CID11687749 have shown an effective attachment to the priming site of viral RdRp, which could lead to replication failure. Moreover, for further safety, health concerns and vaccine development, the suggested compounds of our in silico assessment need further in vitro analysis for future confirmation which will lead towards preclinical trials.

## Supplementary information

**Additional file 1.** Origin and Accession # of recently emerged SARS coronaviruses and viral RdRps.

## Data Availability

All the supported data is included in the manuscript and where applicable hyperlinks are provided. Some of the data has been placed in additional information submitted with this manuscript.

## Abbreviations

AA: amino acids
BBB: blood–brain barrier
DOPE: discrete optimized protein energy
HIA: human intestinal absorption
ICTV: International Committee on Taxonomy of Viruses
MERS: middle east respiratory syndrome
MLE: maximum likelihood estimation
MOE: molecular operating environment
MW: molecular weight
NCBI: National Center for Biotechnology Information
PHEIC: Public Health Emergency of International Concern
RMSD: root-mean-square deviation
ROCT: renal organic cation transporter
SARS: severe acute respiratory syndrome
WHO: World Health Organization
